# Silicon-Vacancy Centers in Ultra-Thin Nanocrystalline Diamond Films

**DOI:** 10.3390/mi9060281

**Published:** 2018-06-02

**Authors:** Stepan Stehlik, Lukas Ondic, Marian Varga, Jan Fait, Anna Artemenko, Thilo Glatzel, Alexander Kromka, Bohuslav Rezek

**Affiliations:** 1 Institute of Physics ASCR, Cukrovarnická 10, Prague 16200, Czech Republic; ondic@fzu.cz (L.O.); varga@fzu.cz (M.V.); fait@fzu.cz (J.F.); artemenko@fzu.cz (A.A.); kromka@fzu.cz (A.K.); rezek@fzu.cz (B.R.); 2 Faculty of Electrical Engineering, Czech Technical University in Prague, Technická 2, Prague 16627, Czech Republic; 3 Department of Physics, University of Basel, Klingelbergstrasse 82, 4056 Basel, Switzerland; thilo.glatzel@unibas.ch

**Keywords:** diamond, color center, nanocrystalline diamond, silicon-vacancy center, Kelvin probe force microscopy, surface photovoltage

## Abstract

Color centers in diamond have shown excellent potential for applications in quantum information processing, photonics, and biology. Here we report the optoelectronic investigation of shallow silicon vacancy (SiV) color centers in ultra-thin (7–40 nm) nanocrystalline diamond (NCD) films with variable surface chemistry. We show that hydrogenated ultra-thin NCD films exhibit no or lowered SiV photoluminescence (PL) and relatively high negative surface photovoltage (SPV) which is ascribed to non-radiative electron transitions from SiV to surface-related traps. Higher SiV PL and low positive SPV of oxidized ultra-thin NCD films indicate an efficient excitation—emission PL process without significant electron escape, yet with some hole trapping in diamond surface states. Decreasing SPV magnitude and increasing SiV PL intensity with thickness, in both cases, is attributed to resonant energy transfer between shallow and bulk SiV. We also demonstrate that thermal treatments (annealing in air or in hydrogen gas), commonly applied to modify the surface chemistry of nanodiamonds, are also applicable to ultra-thin NCD films in terms of tuning their SiV PL and surface chemistry.

## 1. Introduction

Diamond color centers have been attracting considerable attention due to their interesting optical properties [1]. Especially, negatively charged nitrogen (NV^−^) and negatively charged silicon vacancies (SiV^−^) show potential for applications in quantum photonics and sensing. Quantities of interest are magnetic fields, pressure, temperatures, as well as the presence of various biological species. The NV^−^ centers have already been shown to be capable of detecting such quantities [2]. On the other hand, the sensing capabilities of the SiV centers have only been tested for a handful of these quantities. For instance, optical studies have shown that both zero-phonon-line (ZPL) position and bandwidth changes as a function of the surrounding temperature [3,4]. In addition, sensitivity to a magnetic field was demonstrated via the spectral splitting of energy levels at low temperature [5].

The advantage of the SiV^−^ center over the NV^−^ center is that 70% of its light emission is concentrated into the ZPL peaked at around 737 nm [6]. Furthermore, the ZPL of the SiV^−^ center is, even at room temperature, spectrally narrow, which opens up the possibility of measuring tiny changes in the local environment through detection of changes in its optical spectrum.

In order to detect very small changes (e.g., photoluminescence (PL) or spectral position) in the local environment, SiV centers should be positioned in the close vicinity of the diamond’s surface. By accommodating a fabrication process used for NV^−^ center based sensors [7], this could be achieved by ion implantation of Si into the single-crystal diamond followed by thermal annealing and subsequent etching of the surface to reach the SiV center-rich layer. A more straightforward approach that enables creation of <10 nm thin diamond films with SiV centers is a microwave plasma assisted chemical vapor deposition (MW CVD) growth with a source of Si near the grown film [8]. This leads to diamond films with high density of active SiV centers. The limitation of the latter method is that it does not yet enable creation of well spatially separated single centers, a prerequisite for sensing with nanoscale spatial resolution. The sensitivity of the optical centers could be further improved by coupling them with photonic [9,10] or plasmonic [11,12] nanostructures.

If we neglect spin-orbital coupling, the electronic level structure of the SiV^−^ can be simplified by a three-level system: A ground state and a high energy excitation level from which the carriers quickly decay into a light-emitting state [13]. However, even though SiV^−^ centers are intensively studied, a generally-accepted overall picture that would cover the physics behind the light emission from them is not yet available. Namely, the absolute position of the electronic bands within the band structure of diamond [13,14,15], the photoionization process of SiV^−^ centers [16], or even switching between the SiV^−^ and the charge neutral SiV^0^ [17], are still debated topics.

Kelvin probe force microscopy (KPFM) already showed great potential in characterization and distinguishing of nanodiamonds with different surface chemistry [18], and, when combined with surface photovoltage (SPV) measurement, proved to be a useful tool for detection and investigation of color centers such as NV centers in nanodiamonds on nanometer scale [19]. In this work, the sensitivity of the shallow SiV centers to the surface termination as a function of the nanocrystalline diamond (NCD) thin films’ thickness is studied via the changes in the PL intensity and surface potential. By correlation of PL and surface photovoltage (SPV) measurements we demonstrate that decrease of SiV PL intensity of hydrogenated NCD is not caused by the activation of neutral SiV^0^ but rather by activation of non-radiative electronic transitions. This effect is accompanied by detecting relatively strong SPV on hydrogenated NCD due to surface states electron trapping. Further we show ultra-thin NCD surface chemistry modifications by plasma and thermal treatments and corresponding reversibility of the SiV PL intensity with changing the surface termination from oxygen to hydrogen by thermal and plasma treatments.

## 2. Materials and Methods

Two ultra-thin nanocrystalline diamond (NCD) film sets in the range 10–40 nm (NCD1) and 7–16 nm (NCD2) were grown by MW CVD plasma. To achieve continuous ultra-thin NCD films we employed 2 nm detonation nanodiamonds (DNDs) to form uniform seeding layer with ultra-high nucleation density (~1.3 × 10^13^ cm^−2^) and thickness of about 2 nm [8]. Thickness of the NCD films was measured at the Si substrate-NCD film interface by atomic force microscopy (AFM), with thickness corresponding to a mean Z value obtained from the NCD film. The scanning electron microscopy (SEM) images of NCD films were acquired at 15 kV and magnification of 300,000× (MAIA 3, Tescan, Brno, Czech Republic) in the regime of secondary electrons.

On NCD1 films we performed two, and on NCD2 films four, treatments to change surface chemistry of the NCD films. Since the MW CVD growth is finished in pure hydrogen plasma, the as-grown NCD films are hydrogenated (H1-NCD). Subsequent surface treatments involved: oxidation by annealing in air at 450 °C for 30 min (O2-NCD), hydrogenation by annealing in hydrogen gas at 700 °C for 6 h (H3-NCD), and oxidation by oxygen radio-frequency (RF) plasma (O4-NCD). Surface chemistry of 7 and 16 nm NCD2 films after each particular surface treatment was investigated by an X-ray photoelectron spectroscopy (XPS) by means of an XPS spectrometer (Kratos, AXIS Supra, Manchester, UK) equipped with a hemispherical analyzer and a monochromatic Al Kα X-ray source (1486.6 eV). The XPS spectra were acquired from the area of 700 μm × 300 μm with the take-off angle 90°. The survey spectra were recorded with a pass energy of 80 eV, whereas the high-resolution spectrum scans were recorded with a pass energy of 20 eV. Obtained XPS spectra were analyzed using CasaXPS software (Version 2.3.0, Casa Software Ltd., Teignmouth, UK) using linear baseline and Gaussian line shapes of variable widths. The measured survey XPS spectra were calibrated on C 1s peak with binding energy of 285.1 eV that corresponds to sp^3^ phase [20]. XPS peak positions were determined with an accuracy of ±0.2 eV. Photoluminescence data were acquired using an InVia Reflex Renishaw setup. The sample was excited by a HeCd continuous wave 442 nm laser (intensity of 20 mW) focused to a spot of 1 µm on the sample in a direction perpendicular to the sample plane using a 100× objective with NA = 0.9. KPFM was performed in a glovebox filled with dry nitrogen, ensuring inert and humidity-free atmosphere (O_2_ and H_2_O were kept below 0.1 ppm level). Due to significant effect of the adsorbed water on KPFM results from nanodiamond [18], all the samples were annealed at 250 °C for 1 h in the glovebox just prior the KPFM-SPV measurement. The KPFM measurement was carried out in a single pass regime with amplitude modulation (AM), i.e., both topography and contact potential difference (CPD) were acquired simultaneously using the first and second resonance of AFM cantilever using FlexAFM with a C3000 controller (Nanosurf, Liestal, Switzerland), which was used for topography acquisition, and a UHFLI lock-in amplifier (Zurich Instruments, Zurich, Switzerland) served as an external Kelvin controller. The KPFM data were recorded with Pt/Ir5-coated PPP-EFM cantilever (Nanosensors, Neuchâtel, Switzerland) with approximate work function of 4.3 eV [21]. The free oscillation amplitude was 10 nm, setpoint 65%, scan size 1 × 1 μm^2^, scan speed 0.5 Hz, and AC oscillation voltage V_AC_ = 1 V. The cantilever was grounded and both AC oscillation and DC compensation voltage V_DC_ were applied to the sample. The work function was calculated as Φ_sample_ = Φ_tip_ + eV_DC_. The CPD is plotted such that photovoltage lower CPD values correspond to a higher work function (CPD = −eV_DC_). We used an add-on circuit for compensating capacitive crosstalk between KPFM and topography to obtain the most accurate KPFM data [22]. The KPFM data were acquired on plasma hydrogenated (H1-NCD) and air annealing-oxidized (O2-NCD) NCD1 films. SPV measurements [23] were performed simultaneously with KPFM measurements. The light from a supercontinuum laser (NKT Photonics, Birkerød, Denmark) was brought to glovebox via optical fiber to directly illuminate the sample. For excitation of SPV we used red light of 695 nm, 100 nm bandwidth at 100% laser power, which equals to 4.8 mW. SPV values were calculated from the average CPD values of a complete image as SPV = ($\bar{CPD}$_light_ − $\bar{CPD}$_dark_)/e so that lower SPV values correspond to an increase of the work function under illumination induced for example by accumulation of negative charge at the surface.

## 3. Results and Discussion

### 3.1. SiV Centers in Ultra-Thin NCD Films

**Figure 1:**
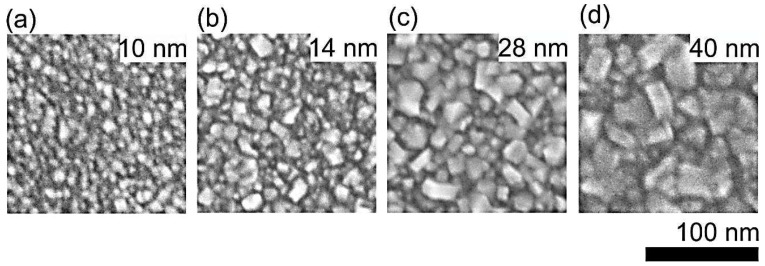
Representative scanning electron microscopy (SEM) images of ultra-thin nanocrystalline diamond (NCD) films having thicknesses of 10 nm (**a**), 14 nm (**b**), 28 nm (**c**), and 40 nm (**d**).

Figure 1 shows representative SEM images of as-grown ultra-thin NCD1 films with thickness 10 nm; RMS = 2.7 nm (a), 14 nm; RMS = 3.4 nm (b), 28 nm; RMS = 4.4 nm (c), and 40 nm; RMS = 6.7 nm (d). Thickness and RMS determination of similar NCD films by means of AFM has been described recently [8]. The SEM images show morphology development from relatively smooth 10 nm NCD film predominantly consisting of sub <10 nm diamond nanocrystals to rougher 40 nm NCD films consisting of larger yet still sub-40 nm diamond nanocrystals.

**Figure 2:**
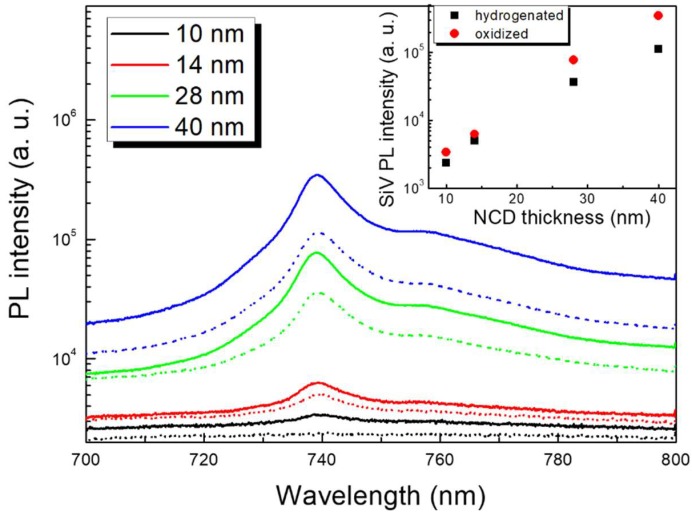
Semi-log plots of photoluminescence (PL) spectra showing shallow silicon vacancy (SiV) PL evolution of plasma-hydrogenated H1-NCD1 films (dotted lines) and air-annealed, i.e., oxidized, O2-NCD1 films (full lines) in dependence on increasing NCD film thickness. The inset shows SiV PL intensity in semi-log scale as a function of NCD film thickness and the NCD surface chemistry.

Figure 2 shows PL data acquired on the H1-NCD1 (dotted lines) and O2-NCD1 (full lines) ultra-thin NCD1 films. All the samples (except the 10 nm H1-NCD1) exhibit a PL peak at ~739 nm which is assigned to ZPL of negatively charged SiV center, i.e., SiV^−^ in diamond [24]. Figure 2 shows that SiV PL intensity depends on the thickness as well as on the NCD film surface chemistry. For both H1-NCD1 and O2-NCD1 the SiV PL intensity increases non-linearly with increasing thickness of the films as better shown in the inset of Figure 2. Please note the log-scale of the PL for better clarity. This non-linear steep increase may be caused either by increase of SiV concentration with increasing thickness or by better PL excitation/extraction from thicker and rougher NCD films. Unfortunately, vertical distribution of SiV in our NCD films, as well as an average concentration of PL active SiV centers is at the moment unknown, but it may be expected that as soon as the NCD film is closed at thickness of 5–10 nm the Si substrate itself no longer provides Si atoms and only the bare Si wafers placed in the plasma serve as the Si source. This means that the rate of SiV centers incorporation (and thus the SiV concentration) should stabilize for >10 nm NCD films, which is supported by a nearly linear dependence of the NCD thickness on the deposition time. The last factor to consider is a non-radiative energy transfer from excited SiV centers to the substrate [25]. It has been shown [26] that photoluminescence of organic dyes is strongly quenched for short distances (<20 nm) to the Si substrate. Similar mechanism may quench the SiV PL from ultra-thin diamond layers. To elucidate which effect dominates is a future task and goes beyond the scope of this manuscript.

In accordance with previous results [8] the SiV PL intensity depends also on the surface chemistry of the NCD films. While the SiV PL is not detectable in the thinnest 10 nm H1-NCD1 film, it appears in the same sample after the surface becomes oxidized after annealing in air oxidation treatment. The O2-NCD1 films have generally higher SiV PL than the H1-NCD1 counterparts. To explain this behavior two processes can be considered. First, in analogy to NV centers [27], hydrogen/oxygen diamond surface termination may control occupancy of two SiV charge states (SiV^−^ and SiV^0^), i.e., the lower SiV PL of H-NCD samples could be due to change of near-surface SiV^−^ to SiV^0^ which emits PL at 946 nm. However, we did not observe any PL peak at 946 nm at temperature of 77 K with 785 nm excitation (data not shown). Therefore, we exclude this mechanism here. The second mechanism to consider is a non-radiative transition of the SiV^−^ centers near the H-NCD surface induced by a specific electronic arrangement at H-NCD surface. To confirm this hypothesis, we performed KPFM/SPV measurements.

**Figure 3:**
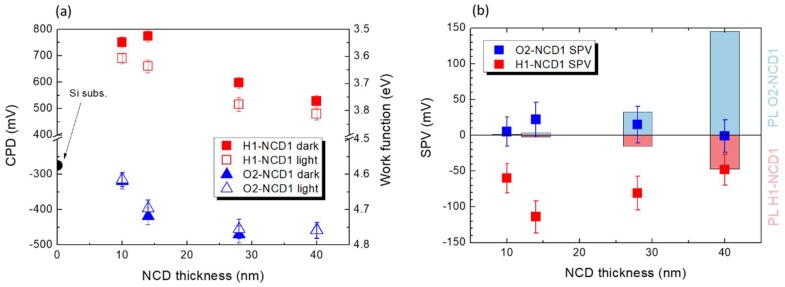
Contact potential difference (CPD) dependence on NCD1 thickness, surface chemistry, and illumination (**a**). Surface photovoltage (SPV) dependence on NCD thickness and surface chemistry. SiV PL intensity is shown as red (H1-NCD1) and blue (O2-NCD2) bars (**b**).

Figure 3a shows evolution of contact potential difference as function of NCD1 film thickness, surface chemistry, and illumination. The CPD value for the p-type Si substrate is also shown. At first, the CPD data shows large work function difference between H1-NCD1 and O2-NCD1 films. Clearly, the largest work function difference (up to 1.08 V) is recorded for the thinnest, sub-20 nm NCD films. As the thickness of the NCD films increases the work function difference slightly decreases down to approximately 1 V for the 40 nm film. These values are well within observed work function variations for hydrogenated and oxidized diamond surfaces [28]. We suppose that the effect of grain boundaries (which is most prominent on the thinnest samples with small grains) can elucidate the lowest work function of the thinnest hydrogenated NCD films, as the grain boundaries are known to decrease energetic barriers for electrons to be extracted from NCD films [29]. Second, nearly all NCD films exhibited a CPD change upon illumination, i.e., SPV, in contrast to Si substrate with no SPV detected. It is remarkable that the generated SPV depends on the NCD thickness and surface chemistry. All the hydrogen-terminated samples showed significant negative SPV, i.e., the work function increased after illumination. In contrast, the oxygen-terminated samples showed less pronounced positive SPV, i.e., the work function slightly decreased after illumination. A closer look at the SPV data indicates the following trend: With increasing thickness the SPV of all the samples at first increases, reaching a maximum at around 15 nm, and then decreases further for thicker samples. The maximal SPV values found for 15 nm NCD samples nicely correlates with the previous results showing that the critical NCD thickness in which the SiV PL can be switched on/off by surface chemistry is about 10 nm [8]. Based on the previous results we may speculate the 15 nm NCD film already has a lot of active SiV centers incorporated, the majority of which are located at a distance not further than 10 nm from the surface and, therefore, are sensitive to the surface chemistry. However, according to this assumption the SPV magnitude should predominantly depend on the incorporated SiV concentration that should stabilize after a few minutes of the CVD process. To explain gradual decrease of SPV in NCD films thicker than 15 nm we propose some kind of resonance energy transfer mechanism between adjacent SiV centers. Considering the resonance transfer mechanism, as soon as the NCD film thickness is greater than 15 nm, the excited, near-surface SiV centers transfer their energy to deeper (not surface affected) SiV centers rather than building up SPV by non-radiative means. The proposed resonance transfer may be similar to Förster resonance energy transfer via dipole-dipole coupling known from non-radiative energy transfer between molecules (which decays strongly with distance ~1/r^6^), or it may be similar to plasmonic effects observed, for instance, during SiV interaction with Au nanoparticles on surface which can reach farther distances of ~10 nm [30].

We use the obtained PL and SPV data to propose a preliminary model which describes the observed PL and SPV surface chemistry and layer thickness dependence. With regard to lower SiV PL and relatively high negative SPV of H-NCD we propose that excited (electron) energy level of near surface (10–15 nm) SiV centers is situated above the Fermi level on the H-NCD surface, which is not pinned and moves towards valence band due to H-induced surface upward band bending [31]. Photo-excited electrons are thus energetically unstable there and become trapped in residual surface states on H-terminated surface and/or grain boundaries [32], instead of the radiative recombination within SiV center. Energetic position of these surface states is around 0.8 eV above the valence band according to our measured work function of about 3.6 eV (see Figure 3a) and expected negative electron affinity (NEA) of H-terminated diamond around −1.1 eV [31]. Probably, no SPV would be generated in perfectly hydrogenated, defect-free monocrystalline diamond according to theoretical calculations [33]. However, the prepared ultra-thin NCD films certainly cannot be considered as a perfect diamond material due to very small diamond grains and significant if not dominant portion of grain boundaries, which represent conductivity barriers and may act as electron traps and give rise to the observed SPV.

The much smaller and opposite polarity SPV observed on O-NCD correlates with higher SiV PL of O-NCD, i.e., radiative transition in SiV occurs and thus SPV is smaller. In addition, on O-NCD, the bands are bent upward but the Fermi level is pinned due to high density of surface states (around 1.8–2.2 eV above the valence band) there that act as hole traps [34]. Thus, the SiV system remains fully below the Fermi level, even near the surface, and SiV PL is higher than near H-terminated surface. Since the positive SPV was recorded on O-NCD, it is likely that some of the photo-generated holes in the SiV system become additionally trapped on the surface states. The hole trapping may be further promoted by the upward surface band banding that helps holes travel to the surface. This somewhat weakens the SiV PL near the surface compared to bulk SiV.

**Figure 4:**
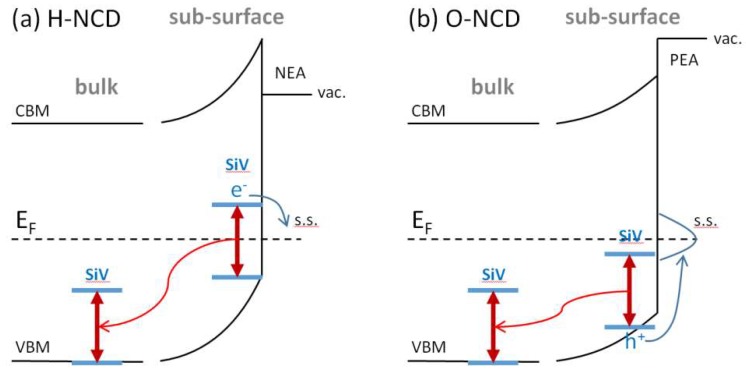
Schematic model of the proposed mechanism for the opposite SPV and its decrease with increasing NCD layer thickness together with increasing PL intensity for the hydrogenated (**a**) and oxidized (**b**) NCD surfaces.

Figure 4 shows schematically the above proposed mechanism of opposite SPV and its decrease with increasing layer thickness together with increasing PL intensity for the hydrogenated and oxidized surfaces. For simplicity, two sketched energy levels of SiV correspond to a ground state and light-emitting state.

### 3.2. Perspectives of Reversible NCD Surface Chemistry Modification by Plasma and Thermal Treatments

**Figure 5:**
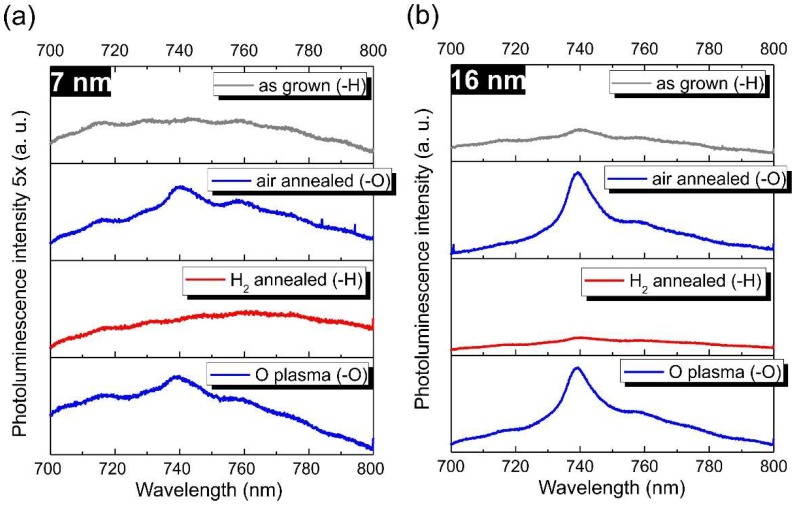
SiV PL data of 7 nm (**a**) and 16 nm (**b**) NCD2 films in dependence on the surface chemistry variation. The specific treatments are mentioned in the legends for clarity.

The PL and CPD/SPV results shown and discussed above were acquired on hydrogen-terminated and oxygen-terminated NCD1 films obtained by hydrogen plasma and annealing in air respectively. The former was achieved by hydrogen plasma applied after the final stage of the NCD growth, which is a common approach for the obtaining of H-NCD films. The latter was achieved by annealing in air at 450 °C for 30 min. This treatment has been well established for oxidation and selective purification of nanodiamonds (NDs) from a non-diamond sp^2^ carbon [35]. The PL results shown here indicate that the same treatment effectively oxidizes also the NCD films. Further we show another two treatments that might be used to effectively control the SiV PL in ultra-thin NCD films and overall reversibility of the applied treatments. Figure 5 shows SiV PL data of 7 nm and 16 nm NCD2 films in dependence on their surface chemistry/treatment. SiV PL increases from hydrogenated (H-plasma) to oxidized (air annealed) NCD2 films similarly as shown in Figure 2 for the NCD1 set. Interestingly, the SiV PL is again reduced for both 7 nm and 16 nm films after they are annealed in hydrogen gas at 700 °C for 6 h. This signalizes change of oxygen surface termination back to hydrogen termination. Again, these hydrogenation conditions are well established for DNDs [18]. It is the reactivity of the DND surface which enables their hydrogenation through a radical reaction [36]. Our PL results indicate that hydrogenation at the same conditions provides well hydrogenated <20 nm ultra-thin NCD films. Since the hydrogen gas dissociates only at very high temperatures (2200 °C), the radical reaction mechanism can also be considered here. In other words, the annealing in hydrogen (700 °C, 6 h) is still able to hydrogenate mostly sub-20 nm diamond nanocrystals such as those in 7 nm and 16 nm NCD films, as shown by XPS data below, and suppress their SiV PL. Finally, the O plasma oxidizes the hydrogenated NCD surface and the SiV PL is restored to a similar level as after the oxidation by annealing in air. This final, low temperature O plasma treatment rules out any possible thermal SiV PL quenching effect during the annealing in hydrogen gas at 700 °C and suggests the surface chemistry nature of the observed reversibility of the SiV PL in the ultra-thin NCD films.

Table 1 summarizes relative atomic concentration of C, O, and Si obtained from high resolution XPS spectra of 7 nm and 16 nm NCD2 films, documenting surface chemistry changes after the particular treatment which are in correlation with the PL data depicted in Figure 5. The XPS data clearly show significant enhancement of O concentration after annealing in air for both 7 nm and 16 nm NCD2 film accompanied by increase of SiV PL. After subsequent annealing in hydrogen gas we observed great (from 10.1 at.% down to 1.8 at.% for 7 nm NCD2) and nearly total (from 9.4 at.% down to 0.5 at.% for 16 nm NCD2) reduction of the oxygen concentration accompanied by drop of SiV PL. Finally, as soon as the NCD surface becomes oxidized by O plasma, the O content returns to similar values as after oxidation by annealing in air.

Due to obvious correlations of Si and O concentrations in 7 nm and 16 nm NCD2 films we ascribe the residual oxygen content in hydrogen gas annealed NCD2 films to Si substrate covered by a native SiO_x,_ rather than to C–O or C=O bonds on the NCD2 film surface. Higher Si concentration recorded for the 7 nm NCD2 film is most probably due to higher pinhole density and overall lower thickness in comparison to the 16 nm NCD2 film. We suppose that observed certain fluctuation of the Si concentration in the 7 nm NCD2 film is related to uncertainty of measurement and processing of XPS spectra, rather than with real Si concentration changes.

## 4. Conclusions

In this work we investigated optoelectronic properties of SiV centers in ultra-thin (7–40 nm) NCD films. We showed that SiV PL is sensitive to NCD surface chemistry, in particular hydrogen-terminated NCD exhibited lower SiV PL intensity than oxygen-terminated NCD. KPFM measurements showed around 1 V lower work function of the hydrogen-terminated than oxygen-terminated NCD. By means of thickness and surface chemistry dependent KPFM/SPV measurements we identified significant positive SPV and less pronounced negative SPV on hydrogen-terminated and oxygen-terminated NCD respectively. This lead us to propose a tentative model ascribing the positive SPV on H-NCD to non-radiative SiV electron transition to surface states (on diamond or in grain boundaries) due to a Fermi level moving below SiV energetic levels on H-NCD surface. On the other hand, overall higher SiV PL of O-NCD compared to H-NCD and negligible SPV found on O-NCD signalizes efficient excitation-emission process without significant electron escape, yet still some holes seem trapped in the surface states. Moreover, for films thicker than 16 nm the SPV amplitude decreases while PL increases for both types of surfaces. We propose that this is related to resonant energy transfer from sub-surface SiV to bulk SiV centers. We demonstrate that thermal treatments commonly applied to surface chemistry modifications of nanodiamonds, such as annealing in air or in hydrogen gas, can be successfully applied also to ultra-thin NCD films to desirably and reversibly modify their surface chemistry and/or SiV photoluminescence. 

## Figures and Tables

**Table 1:** Relative atomic concentration of chemical elements of the 7 and 16 nm NCD2 before and after hydrogenation by annealing in hydrogen gas at 700 °C for 6 h calculated from XPS spectra.

Sample	C, at.%	O, at.%	Si, at.%
7 nm H1-NCD2 (H-plasma; as grown)	93.9	3.9	2.2
7 nm O2-NCD2 (air-annealed)	88.1	10.1	1.8
7 nm H3-NCD2 (H_2_-annealed)	96.1	1.8	2.1
7 nm O4-NCD2 (O-plasma)	87.9	10.3	1.8
16 nm H1-NCD2 (H-plasma; as grown)	96.4	3.4	0.2
16 nm O2-NCD2 (air-annealed)	90.4	9.4	0.2
16 nm H3-NCD3 (H_2_-annealed)	99.2	0.5	0.3
16 nm O4-NCD4 (O-plasma)	91.2	8.5	0.3
